# A randomised, active- and placebo-controlled, three-period crossover trial to investigate short-term effects of the dipeptidyl peptidase-4 inhibitor linagliptin on macro- and microvascular endothelial function in type 2 diabetes

**DOI:** 10.1186/s12933-016-0493-3

**Published:** 2017-01-21

**Authors:** Thomas Jax, Alin Stirban, Arne Terjung, Habib Esmaeili, Andreas Berk, Sandra Thiemann, Robert Chilton, Maximilian von Eynatten, Nikolaus Marx

**Affiliations:** 1 Profil Institut für Stoffwechselforschung GmbH, Hellersbergstr. 9, 41460 Neuss, Germany; 2 Herzzentrum Wuppertal, Universität Witten/Herdecke, Witten, Germany; 3 Staburo GmbH, Munich, Germany; 4 Boehringer Ingelheim Pharma GmbH & Co. KG, Biberach, Germany; 5 Boehringer Ingelheim Pharma GmbH & Co. KG, Ingelheim, Germany; 6 University of Texas Health Science Center at San Antonio, San Antonio, TX USA; 7 RWTH Aachen University, University Hospital Aachen, Aachen, Germany

**Keywords:** Type 2 diabetes, Dipeptidyl peptidase-4 inhibitor, Linagliptin, Sulphonylurea, Endothelial function, Macrovascular, Microvascular, Flow-mediated vasodilation

## Abstract

**Background:**

Studies of dipeptidyl peptidase (DPP)-4 inhibitors report heterogeneous effects on endothelial function in patients with type 2 diabetes (T2D). This study assessed the effects of the DPP-4 inhibitor linagliptin versus the sulphonylurea glimepiride and placebo on measures of macro- and microvascular endothelial function in patients with T2D who represented a primary cardiovascular disease prevention population.

**Methods:**

This crossover study randomised T2D patients (n = 42) with glycated haemoglobin (HbA1c) ≤7.5%, no diagnosed macro- or microvascular disease and on stable metformin background to linagliptin 5 mg qd, glimepiride 1–4 mg qd or placebo for 28 days. Fasting and postprandial macrovascular endothelial function, measured using brachial flow-mediated vasodilation, and microvascular function, measured using laser-Doppler on the dorsal thenar site of the right hand, were analysed after 28 days.

**Results:**

Baseline mean (standard deviation) age, body mass index and HbA1c were 60.3 (6.0) years, 30.3 (3.0) kg/m^2^ and 7.41 (0.61)%, respectively. After 28 days, changes in fasting flow-mediated vasodilation were similar between the three study arms (treatment ratio, gMean [90% confidence interval]: linagliptin vs glimepiride, 0.884 [0.633–1.235]; linagliptin vs placebo, 0.884 [0.632–1.235]; glimepiride vs placebo, 1.000 [0.715–1.397]; *P* = not significant for all comparisons). Similarly, no differences were seen in postprandial flow-mediated vasodilation. However, under fasting conditions, linagliptin significantly improved microvascular function as shown by a 34% increase in hyperaemia area (*P* = 0.045 vs glimepiride), a 34% increase in resting blow flow (*P* = 0.011 vs glimepiride, *P* = 0.003 vs placebo), and a 25% increase in peak blood flow (*P* = 0.009 vs glimepiride, *P* = 0.003 vs placebo). There were no significant differences between treatments in postprandial changes. Linagliptin had no effect on heart rate or blood pressure. Rates of overall adverse events with linagliptin, glimepiride and placebo were 27.5, 61.0 and 35.0%, respectively. Fewer hypoglycaemic events were seen with linagliptin (5.0%) and placebo (2.5%) than with glimepiride (39.0%).

**Conclusions:**

Linagliptin had no effect on macrovascular function in T2D, but significantly improved microvascular function in the fasting state.

*Trial registration* ClinicalTrials.gov identifier—NCT01703286; registered October 1, 2012

**Electronic supplementary material:**

The online version of this article (doi:10.1186/s12933-016-0493-3) contains supplementary material, which is available to authorized users.

## Background

The endothelium plays an important regulatory role in maintaining vascular homeostasis. Impairment of endothelial function (endothelial dysfunction) is an early step in the pathogenesis of atherosclerosis. Endothelial dysfunction is closely associated with the development of diabetic vascular diseases such as nephropathy, neuropathy and retinopathy [1], and is predictive of future cardiovascular events, including cardiovascular mortality [2–4]. Understanding and treating endothelial dysfunction is a major focus in the prevention of macro- and microvascular complications associated with type 2 diabetes (T2D) [1].

Dipeptidyl peptidase (DPP)-4 inhibitors, one of the more recently introduced oral glucose-lowering drug classes, have become widely used as they offer advantages over conventional therapies, in terms of their low risk for hypoglycaemia and neutral effect on body weight [5]. Furthermore, the effects of DPP-4 inhibitors on the macro- and microvascular system are of particular interest because, in addition to their glucose-lowering activity, they may have pleiotropic properties that potentially confer beneficial cardiovascular effects. Several substrates of the DPP-4 enzyme, including the incretin hormone glucagon-like peptide-1 and its metabolites, may directly or indirectly influence cardiovascular function [6, 7].

To date, several large prospective trials have investigated the long-term effects of DPP-4 inhibitors on cardiovascular outcomes in a secondary prevention setting. In patients with advanced diabetes and at high cardiovascular risk, no change in the rates of cardiovascular outcomes was seen [8–10], although potential beneficial effects on the microvasculature such as reduced development and progression of microalbuminuria were reported [9].

Overall, the effects of DPP-4 inhibitors on clinical atherosclerosis remain unclear, especially in patients at an early stage of vascular dysfunction. Previous studies investigating endothelial function have reported heterogeneous effects of DPP-4 inhibitors, both in healthy volunteers [11, 12] and in patients with T2D [13–17]. These heterogeneous results may be attributable to the methods applied and variances in the populations studied. Furthermore, differences in glucose control between study arms could have prevented firm conclusions from being drawn about the pleiotropic effects of DPP-4 inhibition versus effects resulting from the reduction of hyperglycaemia, which is suggested to improve endothelial function [18].

In the present study, we assessed the short-term effects of the DPP-4 inhibitor linagliptin compared with an active comparator (the sulphonylurea glimepiride) and with placebo on measures of macro- and microvascular endothelial function in healthy patients with uncomplicated T2D who were representative of a primary prevention population (i.e., had no history of pre-existing cardiovascular disease).

## Methods

### Study design and patients

This was a randomised, active- and placebo-controlled, three-period crossover, 4-week treatment period, single-centre clinical trial conducted in Germany between November 2012 and January 2014 (ClinicalTrials.gov identifier: NCT01703286; EudraCT number: 2012-003317-33).

The study protocol and amendments were approved by the independent ethics committee of the trial centre. The study was conducted in compliance with the principles of the Declaration of Helsinki, and in accordance with Good Clinical Practice as defined by the International Conference on Harmonisation. All patients provided written informed consent before study initiation.

Consenting patients with a diagnosis of T2D and no diagnosed macro- or microvascular complications, 18‒70 years of age, with a body mass index (BMI) of 25‒35 kg/m^2^ and a glycated haemoglobin (HbA1c) level of ≤7.5%, and stable metformin background therapy (≥1500 mg/day for at least 3 months) were eligible. The main exclusion criteria were: treatment with any glucose-lowering drug (except metformin) within the previous 3 months; any laboratory value or finding of the medical examination (including blood pressure, pulse rate and electrocardiogram) deviating from normal and of clinical relevance; history of cardiovascular disease or major diabetic complication; evidence of a clinically relevant acute concomitant disease; gastrointestinal, hepatic, renal, respiratory, cardiovascular, immunological or hormonal disorders that may influence vascular reactivity or glucose metabolism (except hypertension, hyperlipidaemia and hypothyroidism if treatment was stable for the previous 3 months); gastrointestinal surgery (except appendectomy); diseases of the central nervous system, or psychiatric or neurological disorders; history of relevant orthostatic hypotension, fainting spells or blackouts; chronic or relevant acute infections; history of relevant allergy/hypersensitivity (including allergy to the drug or its excipients); intake of drugs with a long half-life (>24 h) within at least 1 month or less than 10 half-lives of the respective drug prior to administration or during the trial. Other exclusion criteria included participation in another trial with an investigational drug within the previous month, drug or alcohol (>60 g/day) abuse, smoker, blood donation and excessive physical activities (within 1 week prior to administration or during the trial).

### Study procedures

**Fig. 1:**
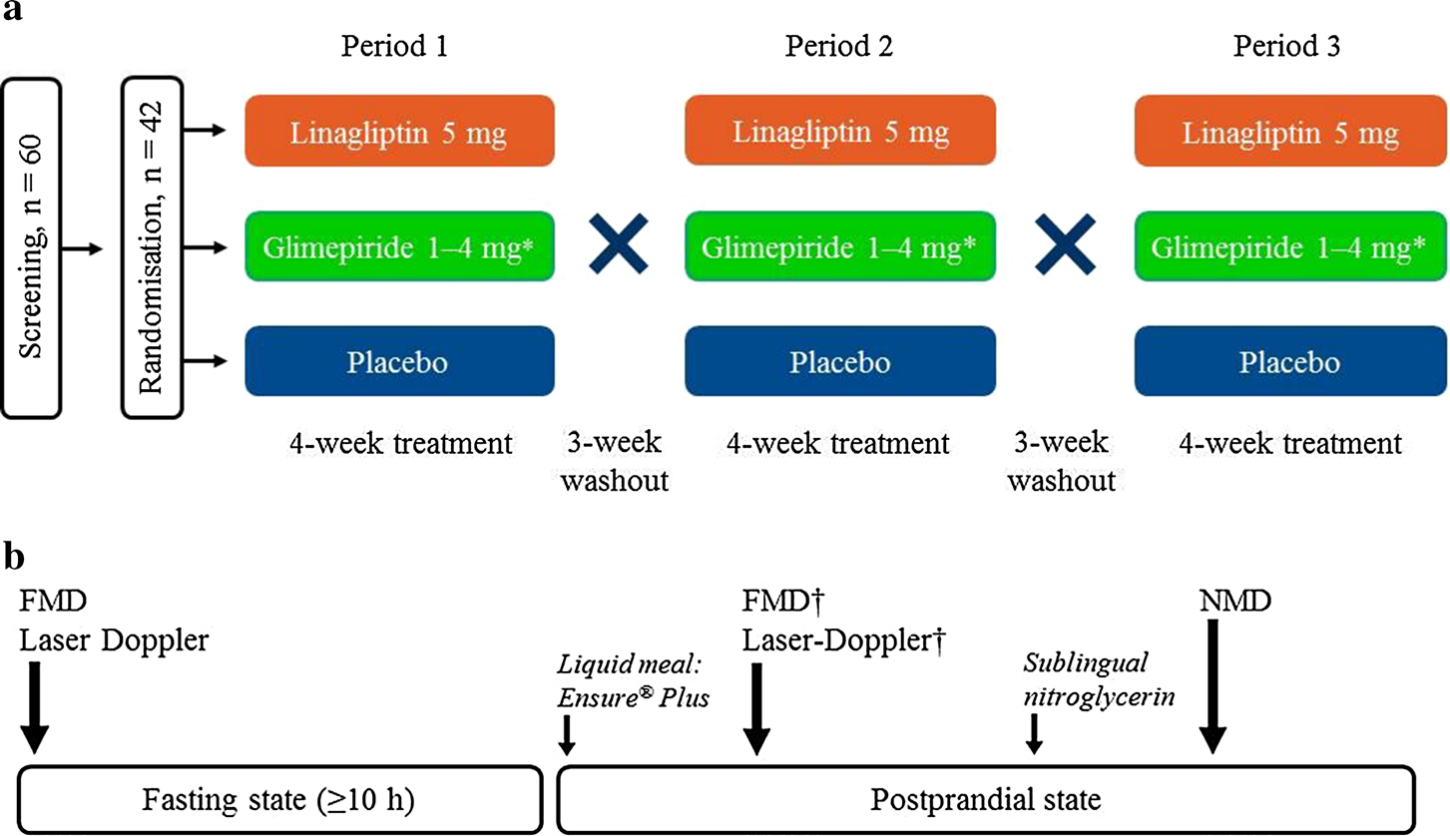
**a** Study design. **b** Vascular assessments on day 1 and day 28 of the 4-week treatment periods. *FMD* flow-mediated vasodilation, *NMD* nitroglycerin-mediated vasodilation. *Glimepiride dose uptitration protocol: initial daily dose of 1 mg for 1 week, uptitrated to 2 mg from week 2; further uptitration to maximum daily dose of 4 mg was allowed if fasting glucose levels were >110 mg/dL (>6.1 mmol/L) at days 14 and 21, and at the investigator’s discretion. ^†^2-h postprandial

After screening, patients received daily placebo (A), glimepiride 1‒4 mg (B), or linagliptin 5 mg (C), in addition to continuing metformin during each 4-week treatment period. There was a 3-week washout between each treatment period to prevent carry-over effects (Fig. 1a). Patients were randomised to one of six treatment sequences according to the Williams square design (ABC, CBA, BCA, BAC, ACB or CAB) such that every patient received each of the three treatments; the block size was six. Randomisation was performed using a validated system which involved a pseudo-random number generator and a supplied seed number. Treatments were masked using a double-blind and double-dummy design.

For the first week, the starting dosage of glimepiride was 1 mg/day. The dosage was then increased to 2 mg/day, and could be uptitrated to a maximum dosage of 4 mg/day if fasting home blood glucose levels were >110 mg/dL (6.1 mmol/L) on days 14 and 21, unless the risk for hypoglycaemia was increased. The dose could be downtitrated at any time to prevent recurrent hypoglycaemic events. No specific rescue drugs were anticipated for the treatment of adverse events and no additional treatment was planned. If adverse events occurred, the patient was to be treated as necessary (as judged by the investigator) and kept under constant supervision.

Metformin monotherapy was the only concomitant glucose-lowering treatment permitted. No other concomitant therapy was allowed, except for statins, angiotensin-converting enzyme (ACE) inhibitors, oral contraceptives and ovary and stable thyroid hormone replacement. Angiotensin receptor blockers were allowed if used within a stable regimen and if they were known to have no impact on nitric oxide metabolism.

### Endpoints

The primary efficacy endpoint was macrovascular endothelial function under fasting conditions, measured as the change from baseline in flow-mediated vasodilation on day 28. Secondary endpoints were macrovascular endothelial function under postprandial (2-h) conditions, measured as the change from baseline in flow-mediated vasodilation on day 28, and macrovascular endothelial-independent vasodilation under postprandial (2-h) conditions (standardised liquid meal), measured as the change from baseline in nitroglycerin-mediated vasodilation on day 28.

Other efficacy endpoints included changes from baseline on day 28 in the following variables: microvascular function measured using laser-Doppler under fasting and postprandial conditions; macrovascular function biomarker levels (soluble P-selectin, E-selectin, von Willebrand factor) under fasting conditions; systemic nitric oxide metabolite levels (nitrates, nitrites) under fasting and postprandial conditions; levels of plasma glucose and insulin; lipid metabolism (levels of triglycerides, low-density lipoprotein [LDL], high-density lipoprotein [HDL], free fatty acids); 24-h continuous blood pressure monitoring.

Endpoints of safety included the frequency and intensity of adverse events. Other safety endpoints were: physical examination; vital signs (systolic and diastolic blood pressure [SBP and DBP], pulse rate); clinical laboratory tests. The following pre-specified adverse events of special interest were reported: hepatic injury (an elevation of aspartate transferase and/or alanine transferase levels of ≥threefold the upper level of normal [ULN] combined with an elevation of total bilirubin of ≥twofold ULN measured in the same blood sample); hypersensitivity reactions (e.g., angioedema, angioedema-like events or anaphylaxis); skin lesions (e.g., exfoliative rash, skin necrosis or bullous dermatitis); renal adverse events (e.g., acute renal failure, a ≥twofold increase in creatinine levels); pancreatitis. Hypoglycaemia was defined as blood glucose levels of ≤70 mg/mL (≤3.9 mmol/L), with or without typical symptoms; severe hypoglycaemia was defined as requiring the assistance of another person to administer resuscitative actions. Version 16.1 of the Medical Dictionary for Regulatory Activities (MedDRA) was used to code adverse events.

### Vascular assessments

At the start (day −1) and end (day 28) of each 4-week treatment period, vascular assessments were performed after fasting for at least 10 h and then 2 h after a liquid meal test (Ensure® Plus drink, Abbott) (Fig. 1b).

The ultrasound procedures for assessing flow-mediated vasodilation of the brachial artery were carried out on the right arm with patients in the supine position (unless there were valid reasons to use the left arm). Measurements were made after a 10-min rest in a quiet dark room at a temperature of approximately 22 °C. A high-resolution ultrasound scanner with a 12.0-MHz linear array transducer was used (General Electric Vivid 7; GE Healthcare, Milwaukee, WI, USA). The brachial artery was scanned longitudinally, just above the antecubital crease. To ensure good reproducibility of repeat measurements, the patient’s anatomic markers and the transducer position were utilised.

To assess endothelium-dependent vasodilation, baseline diameter measurements were obtained. Arterial occlusion was then performed by inflating a forearm blood pressure cuff (12.5 cm wide) to 50 mmHg above the SBP for 5 min. Diameter changes were expressed as the percentage change relative to the mean baseline value. A computer system with automated tracing of echo interfaces and measurements of distances between the wall echoes within a defined section of the brachial artery was used. Images obtained during vascular assessments were digitally acquired and were evaluated offline with a dedicated software tool (Vascular Research Tools 5, Medical Imaging Applications, LLC, USA). Brachial artery diameter was calculated in diastolic frames taken coincidentally with the R wave on the electrocardiogram between 60 and 90 s after cuff deflation. The maximum diameter of these measurements compared with the baseline diameter was used for analysis. Flow-mediated vasodilation was defined as the percentage increase in artery diameter during hyperaemia (100 × [(diameter after hyperaemia − baseline diameter)/baseline diameter]).

After a 10-min rest, endothelial-independent vasodilation was assessed under 2-h postprandial conditions. Brachial artery scans were obtained at baseline and 5 min after administration of sublingual nitroglycerin (0.4 mg glyceryl trinitrate, an exogenous nitric oxide donor). Nitroglycerin-mediated vasodilation was defined as the maximum percentage increase in vessel diameter after nitroglycerin administration.

Microvascular function was assessed using laser-Doppler flowmetry (PF5000; Perimed AB, Järfälla, Sweden) to measure blood flow on the dorsal thenar site of the right hand, which was quantified as arbitrary perfusion units (as laser-Doppler flowmetry cannot measure absolute blood flow). Measurement variables were the pre-ischaemia blood flow (resting blood flow) and maximal post-ischaemia blood flow during reactive hyperaemia (peak blood flow) after 5 min of suprasystolic ischaemia of the forearm (Additional file 1: Figure S1). The reactive hyperaemia reflects the endothelium-dependent vasoreactivity of the microcirculation. Reactive hyperaemia was defined as the area under the curve of blood flow measured continuously over 120 s after cuff release. The parameters described above were calculated using commercially available software (PeriSoft for Windows 2.50; Perimed AB, Järfällä, Sweden).

### Statistical analysis

This was a hypothesis-generating trial and, therefore, all statistical evaluations should be considered descriptive and not inferential. SAS version 9.2 was used for all analyses. The trial was planned to include a total of 42 patients. Allowing for up to six patients to drop out during the trial, N = 36 was used as the sample size for precision calculations. An estimated standard deviation (SD) of approximately 3% for flow-mediated vasodilation measurements was assumed for the trial site (Profil Institut für Stoffwechselforschung GmbH, Neuss, Germany) and, given the sample size of 36 patients, the precision of the two-sided 90% confidence interval (CI) of the flow-mediated vasodilation population effect was calculated to be approximately 1.027 (upper confidence limit/lower confidence limit). For a greater SD of 3.5%, the precision would still be approximately 1.032.

An analysis of variance (ANOVA) model was used for the primary efficacy endpoint. This model included “patients within sequences” as a random effect and “sequence”, “period” and “treatment” as fixed effects. The change from baseline was calculated as the value on day 28 minus the respective value at baseline, and was expressed as the ratio of fasting flow-mediated vasodilation on day 28 to baseline fasting flow-mediated vasodilation. Baseline was the mean of fasting flow-mediated vasodilation values on day −1 across all three treatment periods. For the secondary endpoints and measures of microvascular function, change from baseline on day 28 in postprandial flow-mediated vasodilation and nitroglycerin-mediated vasodilation was analysed with an ANOVA in the same way as the primary endpoint.

Point estimates of the primary and secondary endpoints and their two-sided 90% CIs were reported. For each endpoint, the difference between the expected means was estimated using the difference in the corresponding adjusted means and a two-sided 90% CI based on the *t*-distribution was computed. In addition, the influence of the patient’s age on the primary and secondary endpoints was evaluated using a sensitivity analysis (analysis of covariance, ANCOVA) by additionally adjusting the ANOVA by age.

Efficacy data were analysed in the efficacy set of patients, which included all patients in the treated set (see below) who provided at least one observation for at least one primary, secondary or other efficacy endpoint without important protocol violations relevant for the statistical evaluation of these endpoints.

Safety data were analysed in the treated set of patients, which included all patients who received study medication and took at least one dose of study drug. Safety analyses were summarised descriptively and based on the number of patients with an adverse event (frequency). For laboratory data (including nitric oxide metabolites and vascular biomarkers) and vital signs, the differences from baseline were evaluated using descriptive statistics. Baseline was the last measurement before first trial drug intake in each treatment period.

## Results

### Patient disposition and baseline characteristics

A total of 60 patients were screened; of these, 42 patients were randomised. Three patients discontinued treatment: one patient because of an adverse event (pruritic rash; discontinued in period 2—placebo); two patients withdrew consent (one patient withdrew during period 1—linagliptin; one patient withdrew during washout after period 1—glimepiride). Forty patients entered the placebo and linagliptin treatment periods; 41 patients entered the glimepiride period. All 42 randomised patients were included in the treated set; 41 patients were included in the efficacy set.

Demographics and clinical characteristics at baseline for the treated set of patients are presented in Table 1. Mean age was 60.3 years (SD 6.0, range 46−70 years) and mean BMI was 30.3 kg/m^2^ (SD 3.0, range 25.6–35.0 kg/m^2^). Mean HbA1c was well matched between the three treatment groups (all 7.41% [SD 0.61]). Mean SBP and DBP measurements were approximately 139 and 84 mmHg, respectively. Mean LDL-cholesterol levels were between 2.80 and 2.92 mmol/L; HDL-cholesterol levels were 1.20 mmol/L. The majority of patients (40 patients, 95.2%) had concomitant diagnoses; the most frequent baseline condition was hypertension (23 patients, 54.8%). Most patients (90.5%) reported concomitant therapy use, including ACE inhibitors (28.6%), statins (21.4%) and aspirin (7.1%). There were no differences in fasting and postprandial baseline flow-mediated vasodilation or baseline brachial artery diameter between the groups. Postprandial nitroglycerin-mediated vasodilation was approximately four-fold higher than postprandial flow-mediated vasodilation, suggesting that endothelial smooth muscle cells were sensitive to nitric oxide.

**Table 1:** Patient demographics and clinical characteristics at baseline (treated set)

	Total study population
Patients (n)	42
Age (years)	60.3 (6.0)
Male (%)	66.7
Race (%)	
White	97.6
Asian	2.4
Height (cm)	170.1 (8.6)
Body weight (kg)	87.7 (12.2)
Body mass index (kg/m^2^)	30.3 (3.0)
Hypertension (%)	54.8
Smoking status (%)	
Never smoked	57.1
Ex-smoker	42.9
Alcohol status (%)	
Non-drinker	21.4
Concomitant medication (%)	
Statins	21.4
ACE inhibitors	28.6
Aspirin	7.1
β-Blockers	7.1

	Linagliptin 5 mg	Glimepiride 1–4 mg	Placebo
Patients (n)	40	41	40
HbA1c* (%)	7.41 (0.61)	7.41 (0.61)	7.41 (0.61)
FPG (mg/dL)	135.8 (3.7)	134.5 (3.1)	137.1 (4.2)
Lipids			
LDL cholesterol^†^ (mmol/L)	2.80 (0.83)	2.92 (0.80)	2.80 (0.84)
HDL cholesterol^†^ (mmol/L)	1.20 (0.38)	1.20 (0.36)	1.20 (0.36)
Total cholesterol^†^ (mg/dL)	188.1 (17.1)	190.9 (16.3)	189.5 (16.4)
Triglycerides^†^ (mg/dL)	151.2 (46.9)	153.6 (45.7)	169.9 (134.8)
Brachial artery diameter (FMD)^c^ (mm)			
Fasting, day −1	4.51 (0.56)	4.62 (0.60)	4.60 (0.58)
day 28	4.50 (0.50)	4.52 (0.50)	4.53 (0.51)
2-h postprandial, day −1	4.54 (0.52)	4.57 (0.61)	4.60 (0.64)
day 28	4.49 (0.56)	4.59 (0.60)	4.51 (0.56)
Brachial artery diameter (NMD)^‡^ (mm)			
2-h postprandial, day −1	4.54 (0.55)	4.58 (0.63)	4.62 (0.60)
day 28	4.52 (0.56)	4.60 (0.58)	4.57 (0.48)
SBP^§^ (mmHg)	138.8 (12.9)	139.3 (12.7)	138.8 (12.9)
DBP^§^ (mmHg)	84.2 (6.9)	84.2 (7.0)	84.2 (6.9)
Heart rate^§^ (bpm)	68.4 (9.5)	67.7 (9.8)	68.4 (9.5)

Values are mean (standard deviation) except where indicated

*ACE* angiotensin-converting enzyme, *DBP* diastolic blood pressure, *FMD* flow-mediated vasodilation, *FPG* fasting plasma glucose, *HbA1c* glycated haemoglobin, *HDL* high-density lipoprotein, *LDL* low-density lipoprotein, *NMD* nitroglycerin-mediated vasodilation, *SBP* systolic blood pressure

* Determined at screening visit

^†^Lipids were determined on day −1 of each treatment period

^‡^Post hoc analysis; linagliptin, n = 39; placebo, n = 39 on day 28

^§^Determined at visit 1; linagliptin, n = 39; placebo, n = 39

### Efficacy: vascular assessments

**Fig. 2:**
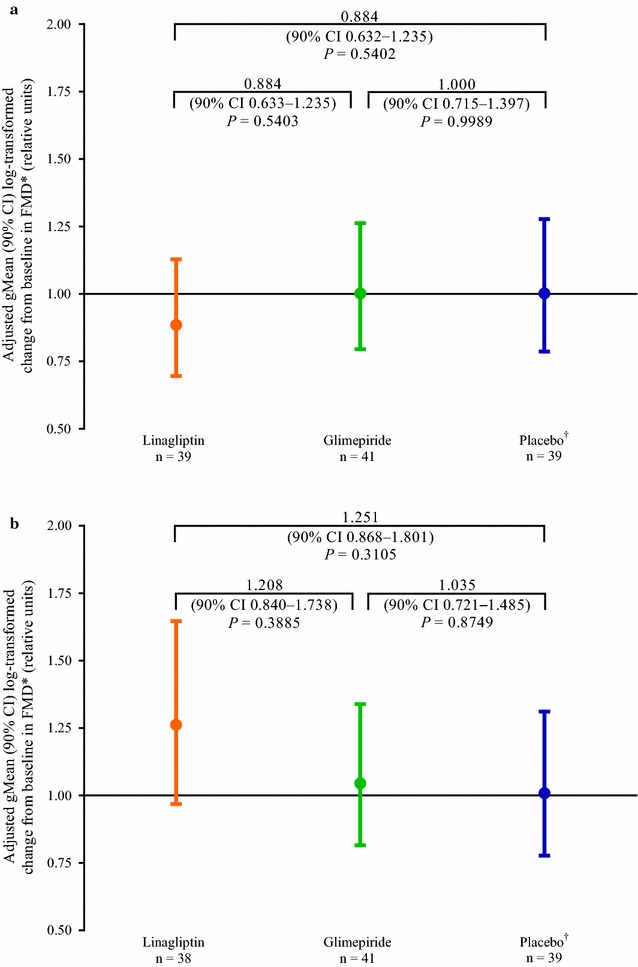
Change from baseline after 28 days between the three treatment groups in brachial endothelial-dependent macrocirculatory function using flow-mediated vasodilation (efficacy set). **a** Fasting. **b** 2-h postprandial. *Ratio of flow-mediated vasodilation on day 28 to flow-mediated vasodilation at baseline. ^†^n = 40 at baseline. *CI* confidence interval, *FMD* flow-mediated vasodilation

For the primary endpoint of change in fasting flow-mediated vasodilation, the adjusted gMean ratios (90% CI) on day 28 to baseline were 0.89 (0.70–1.13) for linagliptin, 1.00 (0.80–1.26) for glimepiride and 1.00 (0.79–1.28) for placebo (Fig. 2a). No statistically significant differences in fasting flow-mediated vasodilation were observed between the treatments (*P* > 0.1 for all comparisons).

For the secondary endpoint of change in postprandial flow-mediated vasodilation, the adjusted gMean ratios (90% CI) on day 28 to baseline were 1.26 (0.97–1.65) for linagliptin, 1.05 (0.82–1.34) for glimepiride and 1.01 (0.78–1.31) for placebo (Fig. 2b). No statistically significant differences in postprandial flow-mediated vasodilation were observed between the treatments (*P* > 0.1 for all comparisons). For the change in postprandial nitroglycerin-mediated vasodilation, the adjusted mean ratios (90% CI) on day 28 to baseline were 1.00 (0.95–1.06) for linagliptin, 1.05 (1.00–1.11) for glimepiride and 0.98 (0.92–1.04) for placebo. Differences between the treatment groups were not statistically significant (*P* > 0.1 for all comparisons). For the primary and secondary endpoints, no effects associated with linagliptin were identified when treatment effects were compared using an ANCOVA model adjusting for age (data not shown).

**Fig. 3:**
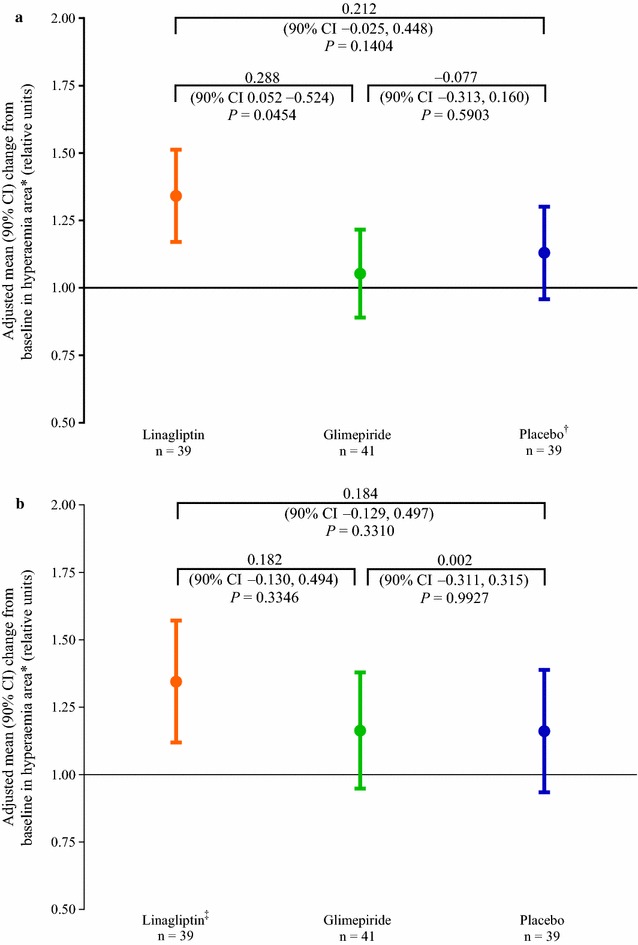
Change from baseline after 28 days between the three treatment groups in endothelial-dependent microcirculatory function using laser-Doppler—hyperaemia area (efficacy set). **a** Fasting. **b** 2-h postprandial. *Ratio of hyperaemia on day 28 to hyperaemia at baseline. ^†^n = 40 at baseline. ^‡^n = 38 at baseline. *CI* confidence interval

**Fig. 4:**
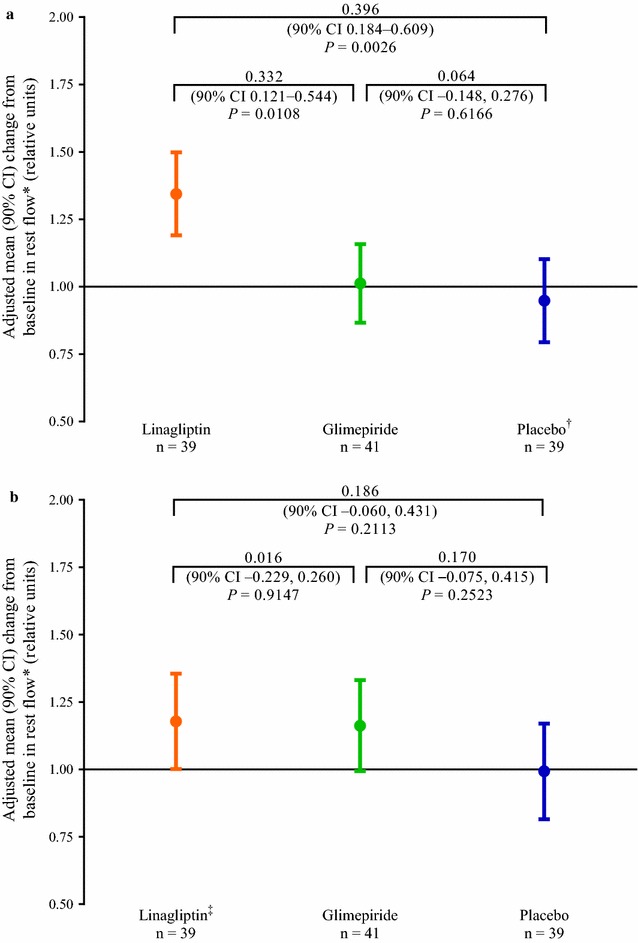
Change from baseline after 28 days between the three treatment groups in endothelial-dependent microcirculatory function using laser-Doppler—resting blood flow (efficacy set). **a** Fasting. **b** 2-h postprandial. *Ratio of resting blood flow on day 28 to resting blood flow at baseline. ^†^n = 40 at baseline. ^‡^n = 38 at baseline. *CI* confidence interval

**Fig. 5:**
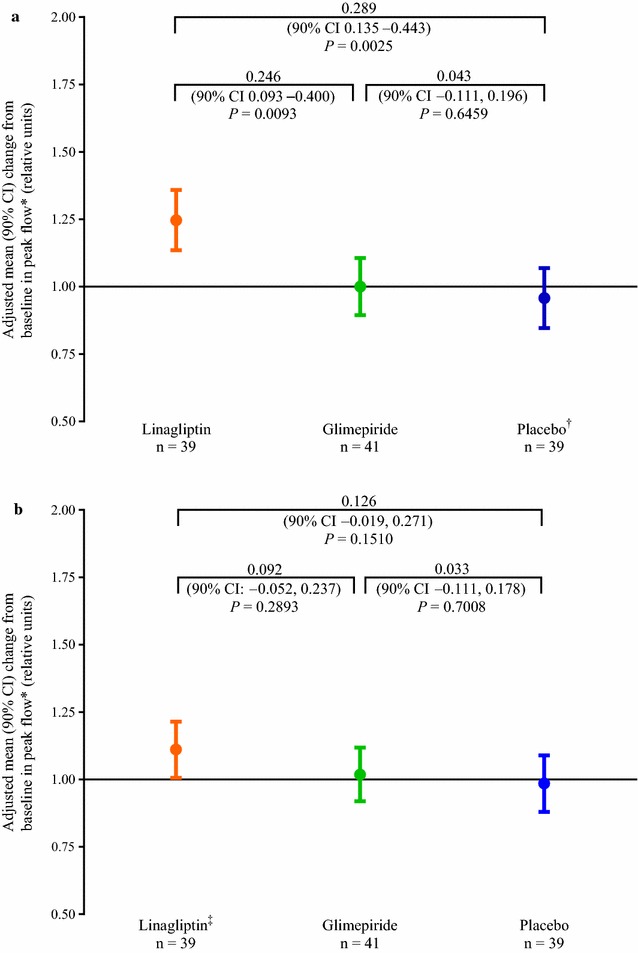
Change from baseline after 28 days between the three treatment groups in endothelial-dependent microcirculatory function using laser-Doppler—peak blood flow (efficacy set). **a** Fasting. **b** 2-h postprandial. *Ratio of peak blood flow on day 28 to peak blood flow at baseline. ^†^n = 40 at baseline. ^‡^n = 38 at baseline. *CI* confidence interval

The changes in fasting and postprandial microcirculatory function, measured using laser-Doppler, are shown in Figs. 3, 4, 5. Under fasting conditions, minimal or no change from baseline in hyperaemia area was detected on day 28 with glimepiride or placebo treatment (adjusted mean [90% CI] ratio: glimepiride, 1.05 [0.89–1.22]; placebo, 1.13 [0.96–1.30]). Hyperaemia area increased by 34% after linagliptin treatment on day 28 compared with baseline (1.34 [1.17–1.51]). This increase was statistically significant for the comparison versus glimepiride (*P* = 0.0454; Fig. 3a). Postprandial hyperaemia area increased on day 28 compared with baseline in all three treatment groups (adjusted mean [90% CI] ratio: linagliptin, 1.35 [1.12–1.57]; glimepiride, 1.16 [0.95–1.38]; placebo, 1.16 [0.94–1.39]). Differences between the treatment groups were not statistically significant (Fig. 3b).

Resting blood flow under fasting conditions on day 28 did not change from baseline with glimepiride or placebo treatment (adjusted mean [90% CI] ratio: glimepiride, 1.01 [0.87–1.16]; placebo: 0.95 [0.79–1.10]). However, fasting resting blood flow increased by 34% with linagliptin treatment (1.34 [1.19–1.50]). This increase was statistically significant for the comparison versus glimepiride (*P* = 0.0108) and versus placebo (*P* = 0.0026) (Fig. 4a). Postprandial resting blood flow increased by 18% with linagliptin and by 16% with glimepiride on day 28 compared with baseline (adjusted mean [90% CI] ratio: linagliptin, 1.18 (1.00–1.36); glimepiride, 1.16 [0.99–1.33]). Postprandial resting blood flow did not change with placebo treatment (0.99 [0.82–1.17]). Differences between the treatment groups were not statistically significant (Fig. 4b).

Peak blood flow under fasting conditions on day 28 did not change from baseline with glimepiride or placebo treatment (adjusted mean [90% CI] ratio: glimepiride, 1.00 [0.89–1.11]; placebo: 0.96 [0.85–1.07]). However, fasting peak blood flow increased by 25% with linagliptin (1.25 [1.14–1.36]). This increase was statistically significant for the comparison versus glimepiride (*P* = 0.0093) and versus placebo (*P* = 0.0025) (Fig. 5a). Postprandial peak blood flow did not change from baseline with glimepiride or placebo on day 28 (adjusted mean [90% CI] ratio: glimepiride, 1.02 [0.92–1.12]; placebo, 0.99 [0.88–1.09]). Postprandial peak blood flow increased by 11% with linagliptin (1.11 [1.01–1.22]). Differences between the treatment groups were not statistically significant (Fig. 5b).

### Effect on levels of nitric oxide metabolites, vascular biomarkers, and cardiovascular risk factors

Compared with baseline, levels of fasting plasma glucose were decreased on day 28 with linagliptin or glimepiride treatment (*P* < 0.001 vs placebo for adjusted mean changes for both), whereas there was no change with placebo (Table 2). The greatest decrease was observed with glimepiride treatment. Similar results were seen with changes in 2-h postprandial plasma glucose levels (*P* < 0.001 vs placebo for adjusted mean changes for both linagliptin and glimepiride). Fasting and postprandial levels of insulin increased with glimepiride treatment, whereas there was little or no change in these levels with linagliptin or placebo (Additional file 1: Table S1).

**Table 2:** Cardiovascular risk factor levels at baseline and after 28 days (efficacy set)

	Linagliptin 5 mg		Glimepiride 1–4 mg		Placebo	
	n = 39*		n = 41^†^		n = 39*	
	Baseline	Unadjusted change from baseline at day 28	Baseline	Unadjusted change from baseline at day 28	Baseline	Unadjusted change from baseline at day 28
Plasma glucose fasting (mg/dL)	135.8 (3.7)	−14.4 (2.4)	134.5 (3.1)	−29.8 (3.6)	137.1 (4.2)	−1.9 (2.0)
Plasma glucose 2-h postprandial (mg/dL)	179.8 (5.5)	−19.7 (4.9)	178.7 (5.9)	−37.3 (4.7)	183.3 (6.8)	0.2 (3.5)
Triglycerides fasting (mmol/L)	1.61 (0.10)	−0.26 (0.08)	1.64 (0.09)	−0.23 (0.10)	1.81 (0.24)	0.07 (0.10)
Triglycerides 2-h postprandial (mmol/L)	1.78 (0.12)	−0.32 (0.06)	1.81 (0.10)	−0.21 (0.08)	1.95 (0.18)	0.02 (0.07)
LDL-cholesterol fasting (mmol/L)	2.80 (0.13)	0.00 (0.06)	2.92 (0.13)	0.04 (0.06)	2.80 (0.13)	0.00 (0.06)
LDL-cholesterol 2-h postprandial (mmol/L)	2.63 (0.12)	0.01 (0.05)	2.76 (0.12)	0.03 (0.06)	2.64 (0.13)	0.02 (0.05)
HDL-cholesterol fasting (mmol/L)	1.20 (0.06)	0.01 (0.02)	1.20 (0.06)	0.03 (0.02)	1.20 (0.06)	0.00 (0.02)
HDL-cholesterol 2-h postprandial (mmol/L)	1.13 (0.06)	0.02 (0.02)	1.13 (0.05)	0.03 (0.02)	1.15 (0.05)	0.01 (0.01)
Day SBP (mmHg)	136.9 (1.9)	−0.1 (0.9)	136.0 (2.1)	1.9 (0.9)	135.7 (2.1)	0.2 (1.2)
Night SBP (mmHg)	124.5 (2.1)	−0.8 (1.2)	126.2 (2.0)	−0.2 (1.1)	124.5 (2.0)	−0.1 (1.3)
24-h SBP (mmHg)	134.3 (1.8)	−0.3 (0.9)	134.1 (2.0)	1.5 (0.9)	133.2 (2.0)	0.1 (1.1)
Day DBP (mmHg)	81.7 (1.3)	0.3 (0.5)	81.0 (1.4)	0.4 (0.6)	80.8 (1.5)	0.3 (0.6)
Night DBP (mmHg)	71.9 (1.4)	−0.7 (0.8)	73.3 (1.5)	−0.2 (0.6)	72.4 (1.4)	−0.4 (0.7)
24-h DBP (mmHg)	79.7 (1.3)	0.1 (0.5)	79.5 (1.4)	0.2 (0.5)	79.1 (1.5)	0.1 (0.6)
Day heart rate (bpm)	78.5 (1.6)	2.7 (1.1)	77.8 (1.6)	1.8 (0.8)	77.4 (1.7)	0.5 (0.9)
Night heart rate (bpm)	68.9 (1.6)	2.0 (0.9)	69.1 (1.6)	1.9 (0.8)	69.3 (1.5)	0.4 (0.7)
24-h heart rate (bpm)	76.5 (1.6)	2.4 (1.0)	76.0 (1.6)	1.7 (0.7)	75.9 (1.6)	0.4 (0.8)

Data are mean (standard error)

*DBP* diastolic blood pressure, *HDL* high-density lipoprotein, *LDL* low-density lipoprotein, *SBP* systolic blood pressure, *Day* 07:00 to 23:00 h; *night* 23:00 to 07:00 h

* Some values at baseline are based on n = 40

^†^ Some values at day 28 are based on n = 40

Levels of fasting and 2-h postprandial triglycerides decreased with linagliptin or glimepiride treatment, whereas there was no change with placebo (Table 2). No changes from baseline were observed in LDL- or HDL-cholesterol levels under fasting or postprandial conditions (Table 2). Fasting free fatty acid levels decreased with glimepiride treatment but not with linagliptin or placebo treatment (Additional file 1: Table S1). There was little or no change in postprandial free fatty acid levels across the treatment groups.

No changes in day or night mean SBP or DBP were detected after 28 days of placebo or linagliptin treatment. A slight increase in day and 24-h mean SBP was observed with glimepiride treatment after 28 days (Table 2). Both day and night mean heart rate increased by approximately 2 bpm after 28 days of linagliptin or glimepiride treatment. There was no change from baseline in mean heart rate with placebo (Table 2).

Changes in the levels of nitric oxide metabolites and vascular biomarkers under fasting or postprandial conditions are included in Additional file 1: Tables S2 and S3. At the end of the study, no significant changes from baseline in fasting or postprandial nitrite and nitrate levels were seen across the treatment groups. Similarly, no significant changes from baseline were detected in the fasting levels of P-selectin, E-selectin or von Willebrand factor.

### Safety

A summary of adverse events is presented in Table 3. Overall, fewer patients had treatment-emergent adverse events with linagliptin (11 patients, 27.5%) than with glimepiride (25 patients, 61.0%) or placebo (14 patients, 35.0%). No adverse events of severe intensity were reported. The frequency of patients with adverse events deemed drug-related by the investigator was highest with glimepiride (18 patients, 43.9%). Adverse events leading to discontinuation of the trial drug were reported by 1 patient (2.5%) receiving placebo. Adverse events of special interest were reported by 2 patients (4.9%) receiving glimepiride and by 4 patients (10.0%) receiving placebo. There were no deaths or other serious adverse events during the treatment phases of the trial.

**Table 3:** Summary of adverse events (treated set)

	Linagliptin 5 mg	Glimepiride 1–4 mg	Placebo
Patients (n)	40	41	40
Any adverse events (%)	27.5	61.0	35.0
Severe adverse events (%)	0.0	0.0	0.0
Drug-related adverse events (%)	17.5	43.9	10.0
Other significant adverse events (according to ICH E3) (%)	0.0	2.4	2.5
Adverse events leading to discontinuation of trial medication (%)	0.0	0.0	2.5
Adverse events of special interest* (%)	0.0	4.9	10.0
Serious adverse events (%)	0.0	0.0	0.0

MedDRA version 16.1 used for reporting

*ICH* International Conference on Harmonisation (E3 guideline)

* Pre-specified adverse events of special interest: hepatic injury, hypersensitivity reactions, skin lesions, renal adverse events and pancreatitis

**Fig. 6:**
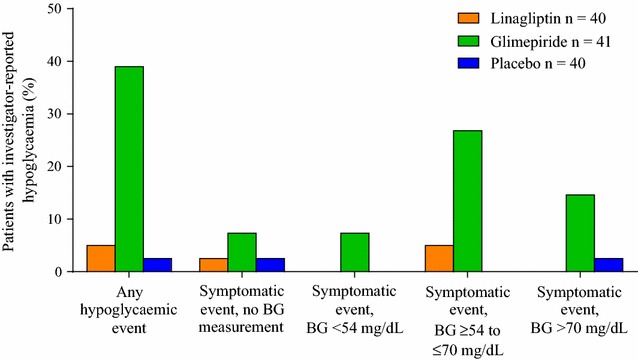
Frequency of patients with investigator-reported hypoglycaemia (treated set). *BG* blood glucose

Investigator-reported hypoglycaemia occurred in 16 patients (39.0%) on glimepiride, 2 patients (5.0%) on linagliptin, and 1 patient (2.5%) on placebo (Fig. 6). Most hypoglycaemic adverse events were symptomatic, with blood glucose levels between ≥3.0 and ≤3.9 mmol/L (≥54 and ≤70 mg/dL), or >3.9 mmol/L (>70 mg/dL). There were no cases of severe hypoglycaemia.

## Discussion

This exploratory clinical trial was the first to compare the effects of DPP-4 inhibition with sulphonylurea treatment and placebo on fasting and postprandial macro- and microvascular endothelial function. The study was designed to assess the glucose-dependent and glucose-independent vascular effects of linagliptin in patients with T2D who were otherwise healthy and who were representative of a primary prevention cardiovascular disease population. As expected, both linagliptin and glimepiride reduced fasting and postprandial plasma glucose levels after 28 days of treatment. Similarly, the safety findings were consistent with the known safety profiles of linagliptin and glimepiride. The overall frequency of adverse events was highest with glimepiride, mainly driven by the comparatively large number of hypoglycaemic episodes in the glimepiride group.

### Effects on macrovascular function

Linagliptin or glimepiride treatment for 28 days did not significantly affect fasting or postprandial macrovascular endothelial function, as measured using flow-mediated vasodilation of the brachial artery. This finding is supported by the lack of change in the levels of several markers of macrovascular endothelial function (nitric oxide metabolites, P-selectin, E-selectin, von Willebrand factor). High-dose nitroglycerin was used as an exogenous nitric oxide donor to determine the maximum obtainable vasodilator response of the brachial artery. Because nitroglycerin-mediated vasodilation was substantially higher than flow-mediated vasodilation in the postprandial state in all treatment groups, we can exclude that the lack of vasodilatory response after shear stress with flow-mediated vasodilation was because of nitric oxide-insensitivity of the vascular smooth muscle cells.

Based on existing evidence that acute reductions in hyperglycaemia may lead to improvements in endothelial function in T2D [19, 20], it is surprising that amelioration of macrovascular endothelial function was not observed with linagliptin or glimepiride in the present study. Possible explanations for this finding include the fact that the patients had relatively well controlled HbA1c levels (7.4%) and, therefore, the effects of glucotoxicity may not yet be contributing to endothelial dysfunction. In addition, the patients had uncomplicated T2D and no clinical indications of atherosclerosis, representing a population at a very early stage of diabetes-related vascular dysfunction and, in this state, linagliptin treatment may have little or no impact on macrovascular endothelial function compared with patients with more advanced T2D and vascular dysfunction. The neutral effect in this primary prevention cardiovascular disease population is consistent with a recent pooled analysis of randomised clinical trials of linagliptin that found no increase or decrease in cardiovascular event rates [21].

Other clinical studies evaluating the effects of DPP-4 inhibition on endothelial function in patients with T2D have reported heterogeneous results. Vildagliptin improved endothelial-dependent vasodilation in a 4-week trial in 16 patients with T2D [16]. Several studies of sitagliptin have shown beneficial effects on endothelial function in patients with T2D [17, 22], including patients with coronary artery disease [14, 23]. In contrast, two separate 6-week studies by Ayaori et al, showed that both sitagliptin and alogliptin significantly reduced endothelial function as measured by flow-mediated vasodilation in 24 patients with T2D [13], while in a 12-week study in 42 patients with T2D (or impaired glucose tolerance) and acute coronary syndromes, sitagliptin did not affect endothelial function (measured using the reactive hyperaemia index) [24]. Sitagliptin also had no effect on macrovascular endothelial function measured by flow-mediated vasodilation and nitroglycerin-mediated vasodilation in a two-year study in T2D patients [25]. However, alogliptin slowed the progression of atherosclerosis (measured using intima media thickness of the carotid artery) in a 24-month study in 314 T2D patients with no apparent cardiovascular disease [26]. The inconsistent findings across these studies may be attributed to differences in the methodologies used to assess the changes in endothelial function, the impact of variations in trial durations, patient populations, background medications or the heterogeneous chemical nature of DPP-4 inhibitors, for example [27].

Three large prospective trials in predominantly secondary prevention populations have shown that several members of the DPP-4 inhibitor class neither increased nor decreased the risk of cardiovascular events versus placebo [5–7]. An ongoing clinical trial (CAROLINA®; NCT01243424) will investigate differences in the effects on cardiovascular outcomes between linagliptin and glimepiride in patients with early T2D with mainly no established cardiovascular complications [28].

### Effects on microvascular function

The noteworthy finding of the present study was that linagliptin improved measures of microvascular function in the fasting state—hyperaemia area, resting blood flow, peak blood flow—as determined after 28 days using laser-Doppler blood flow assessments. Although the improvement in hyperaemia area compared with placebo was not significant, this may reflect either high inter-day variability in resting blood flow [29] or type 2 error. Despite greater improvements in glycaemic control compared with linagliptin, no changes in microvascular function were detected with glimepiride, suggesting that linagliptin may exert beneficial effects on microvascular blood flow independent of glucose control. No effect was seen on postprandial microvascular function with either linagliptin or glimepiride. One explanation for this latter finding could be that postprandial endothelial dysfunction is triggered by different mechanisms than fasting endothelial dysfunction (e.g., hyperglycaemia, hyperinsulinaemia, hypertriglyceridaemia or food toxins leading ultimately to increased oxidative stress [30–32]). Therefore, pleiotropic effects beyond metabolic changes may be more prominent in the fasting than in the postprandial state.

The improvements in microvascular function seen in the present study are consistent with experimental studies using animal models, which have shown that linagliptin has vasodilatory, anti-oxidant and anti-inflammatory properties, and can reduce atherosclerosis, independent of lowering glucose levels [33–35]. Preliminary clinical trials have also shown that linagliptin has the potential to modify microvascular function. In a randomised placebo-controlled trial in 43 non-diabetes patients with a history of arterial hypertension, retinal microvascular blood flow was significantly improved after 12 weeks’ treatment with linagliptin [36]. No difference in the effect on glycaemic control was seen between the treatment groups. Similarly, linagliptin was shown to normalise increased renal endothelial function after 6 weeks’ treatment in a randomised placebo-controlled trial in 62 patients with T2D [37]. Most recently, a randomised placebo-controlled, parallel-group study found that 12 weeks of treatment with linagliptin had no effect on macrovascular function, as measured by flow-mediated vasodilation and nitroglycerin-mediated vasodilation, but exhibited a trend towards improving microvascular function [38]. Taken together, this evidence suggests that linagliptin may improve microvascular function, which supports the results from several pooled analyses of phase III clinical trial data showing that linagliptin significantly reduced clinically relevant kidney disease endpoints in patients with T2D [39, 40]. Based on other clinical studies, it is unclear if improving microvascular function is a class effect of DPP-4 inhibitors: although vildagliptin improved retinal microvascular blood flow [41], saxagliptin reduced retinal blood flow [15], and sitagliptin had no effect on skin microvascular function [42].

We propose several explanations that may account for the discordant effects of linagliptin on macro- and microvascular function seen in our study. Evidence from the UK Prospective Diabetes Study suggests that microvascular complications emerge at a much earlier stage in T2D than macrovascular changes [43], and therefore, it is possible that the effects of linagliptin treatment on endothelial function may be minimal or strictly limited to the microvasculature in the present study given that the patient population had relatively uncomplicated T2D. In addition, the regulation of endothelial-specific processes may vary between macro- and microvascular beds; for example, preclinical studies have shown a greater expression of the DPP-4 enzyme in the microvasculature than in the macrovascular bed [44], a finding which suggests a more prominent role for DPP-4 in microvascular endothelial function. This discordant effect on endothelial function seen in our study is supported by the results from a study in 39 patients who had similar clinical characteristics to those in our study. In patients with T2D and no cardiovascular disease (but with mild diastolic dysfunction), microvascular function was improved after 4 months of oral glucose-lowering therapy, whereas macrovascular function remained unchanged [45]. Taken together, this evidence suggests that the microvasculature may benefit predominantly from DPP-4 inhibition in patients with early signs of vascular disease. Whether these benefits will extend to patients with albuminuria or more advanced renal microvascular disease is currently being evaluated in two prospective clinical trials of linagliptin—MARLINA-T2D™ (NCT01792518) and CARMELINA® (NCT01897532), respectively.

## Limitations

This exploratory study has several strengths and limitations. It was a single-centre study conducted in a relatively low number of patients; however, the study was strengthened by its cross-over design and by the inclusion of both active and placebo controls. The duration of treatment was only 4 weeks, which may be insufficient time for durable changes in endothelial function to occur in this particular study population. Patients’ use of additional medications (e.g., statins, ACE inhibitors) may have affected endothelial function. A post hoc subgroup analysis found no significant differences in changes in flow-mediated dilation, microvascular function, blood pressure or heart rate between patients taking ACE inhibitors (n = 12) and those not taking such medications (n = 30) (data not shown); however, the low patient numbers in these subgroups preclude firm conclusions on the potential confounding influence of ACE inhibitors. Assessment of microcirculation was not the primary or secondary endpoint; a large number of endpoints were assessed, leaving the possibility that the findings are because of an inflated alpha error. The study did not evaluate the mechanism(s) by which linagliptin may improve microvascular function. Finally, the mean glimepiride dose taken during the study is not known, as the case report form used did not capture the number of tablets taken.

## Conclusions

In patients with uncomplicated T2D and no clinical indications of macro- or microvascular complications, linagliptin but not glimepiride improved microvascular endothelial function in the fasting state. The effect appeared to be independent of glucose lowering and was abolished by postprandial hyperglycaemia. Macrovascular function was not affected by either linagliptin or glimepiride treatment. These results suggest that linagliptin may improve microvascular endothelial function during the early stages of diabetic vascular disease.

## Additional file

**Additional file 1.** Supplementary material.

## Abbreviations

ACE: angiotensin-converting enzyme
ANCOVA: analysis of covariance
ANOVA: analysis of variance
BMI: body mass index
CI: confidence interval
DBP: diastolic blood pressure
DPP-4: dipeptidyl peptidase-4
HbA1c: glycated haemoglobin
HDL: high-density lipoprotein
LDL: low-density lipoprotein
MedDRA: Medical Dictionary for Regulatory Activities
SBP: systolic blood pressure
SD: standard deviation
T2D: type 2 diabetes
ULN: upper limit of normal
