# Supplementary material for: A randomised, active- and placebo-controlled, three-period crossover trial to investigate short-term effects of the dipeptidyl peptidase-4 inhibitor linagliptin on macro- and microvascular endothelial function in type 2 diabetes
Source: Cardiovasc Diabetol. 2017 Jan 21;16:13. doi: 10.1186/s12933-016-0493-3 (PMC5251248; doi:10.1186/s12933-016-0493-3)
Supplement: Supplementary file 1 — Additional file 1. Supplementary material. [file 12933_2016_493_MOESM1_ESM.docx]

**Additional material**

**Figre S1** Example of reactive hyperaemia measurement


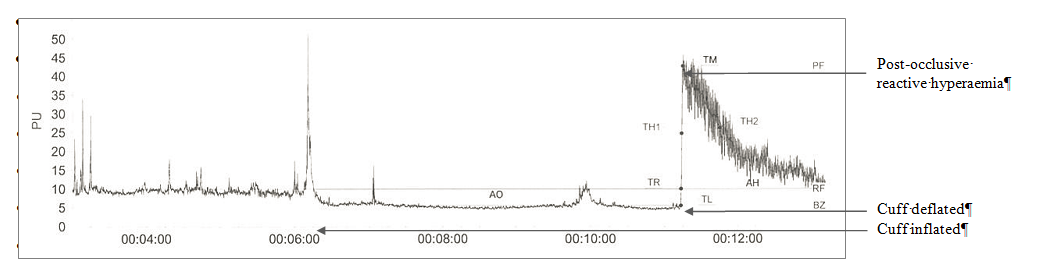


*AO* occlusion area (perfusion units × sec), *AH* hyperaemic area (perfusion units × sec), *BZ* biological zero (perfusion when occluded), *PF* peak flow (highest perfusion value after occlusion is released), *PU* perfusion units, *RF* rest flow (normal perfusion), *TH1* time to half before hyperaemia (time from the release of occlusion until the perfusion value reaches half the value of the peak flow), *TH2* time to half after hyperaemia (time from the release of occlusion until the perfusion value decreases halfway to the rest flow from the peak flow), *TL* time to latency (time until the perfusion increases after the occlusion is released), *TM* time to max (time until the peak flow is reached after the occlusion is released), *TR* time to recovery (time until the rest flow is reached after the occlusion is released)

**Table S1 Insulin and free fatty acid levels at baseline and after 28 days (efficacy set)**

|  | **Linagliptin 5 mg**  **n = 39*** | | **Glimepiride 1–4 mg**  **n = 41** | | **Placebo**  **n = 39*** | |
| --- | --- | --- | --- | --- | --- | --- |
|  | **Baseline** | **Unadjusted change from baseline at day 28** | **Baseline** | **Unadjusted change from baseline at day 28** | **Baseline** | **Unadjusted change from baseline at day 28** |
| Insulin fasting (mU/L) | 12.60 (0.98) | –0.65 (0.57) | 12.30 (0.88) | 6.13 (1.21) | 13.26 (1.22) | –0.25 (0.73) |
| Insulin 2-h postprandial (mU/L) | 41.95 (4.74) | –3.01 (1.72) | 38.60 (3.84) | 7.59 (2.89) | 43.98 (3.88) | –1.01 (2.16) |
| Free fatty acid fasting (mmol/L) | 0.62 (0.04) | 0.02 (0.04) | 0.61 (0.03) | –0.13 (0.03) | 0.56 (0.03) | –0.02 (0.02) |
| Free fatty acid 2-h postprandial (mmol/L) | 0.20 (0.02) | 0.01 (0.01) | 0.20 (0.01) | –0.02 (0.01) | 0.19 (0.01) | 0.01 (0.01) |

Data are mean (standard error)

*Some values at baseline are based on n = 40

**Table S2 Nitric oxide metabolite levels at baseline and after 28 days (efficacy set)**

|  | **Linagliptin 5 mg**  **n = 39** | | **Glimepiride 1–4 mg**  **n = 41** | | **Placebo**  **n = 39*** | |
| --- | --- | --- | --- | --- | --- | --- |
|  | **Baseline** | **Unadjusted change from baseline at day 28** | **Baseline** | **Unadjusted change from baseline at day 28** | **Baseline** | **Unadjusted change from baseline at day 28** |
| Nitrite fasting (µmol/L) | 1.11 (0.05) | –0.03 (0.02) | 1.10 (0.04) | 0.04 (0.04) | 1.10 (0.04) | 0.00 (0.02) |
| Nitrite 2-h postprandial (µmol/L) | 1.13 (0.05) | 0.02 (0.02) | 1.08 (0.04) | 0.00 (0.02) | 1.08 (0.04) | 0.02 (0.02) |
| Nitrate fasting (µmol/L) | 36.09 (1.76) | –0.48 (1.44) | 36.58 (1.85) | 2.38 (2.87) | 36.13 (1.60) | –0.51 (1.92) |
| Nitrate 2-h postprandial (µmol/L) | 32.07 (1.54) | –0.50 (1.10) | 32.48 (1.54) | 1.92 (2.28) | 32.18 (1.31) | –0.36 (1.43) |

Data are mean (standard error)

*Baseline values are based on n = 40

**Table S3 Fasting vascular biomarkers levels at baseline and after 28 days (efficacy set)**

|  | **Linagliptin 5 mg**  **n = 39*** | | **Glimepiride 1–4 mg**  **n = 41*** | | **Placebo**  **n = 39*†** | |
| --- | --- | --- | --- | --- | --- | --- |
|  | **Baseline** | **Unadjusted change from baseline at day 28** | **Baseline** | **Unadjusted change from baseline at day 28** | **Baseline** | **Unadjusted change from baseline at day 28** |
| P-selectin (μg/L) | 87.61 (3.22) | 0.37 (1.47) | 84.67 (3.05) | –0.20 (1.36) | 86.73 (3.29) | 1.39 (1.43) |
| E-selectin (μg/L) | 45.66 (2.54) | –1.33 (1.04) | 43.95 (2.36) | 0.84 (0.94) | 44.12 (2.45) | –1.13 (0.85) |
| Von Willebrand factor (%) | 106.24 (8.00) | –3.94 (6.09) | 113.66 (8.72) | 6.51 (6.33) | 97.66 (9.07) | 1.18 (5.04) |

Data are mean (standard error)

*Baseline values for von Willebrand factor are based on n = 38 for linagliptin and placebo; for glimepiride, day 28 value is based on n = 40

†Baseline values for P-selectin and E-selectin are based on n = 40
